# Performance Study of Diamond Powder-Filled Sodium Silicate-Based Thermal Conductive Adhesives

**DOI:** 10.3390/ma16113937

**Published:** 2023-05-24

**Authors:** Ming Chen, Zhihao Zhou, Xu Wang, Yangchun Zhao, Yongmin Zhou

**Affiliations:** College of Materials Science and Engineering, Nanjing Tech University, Nanjing 211816, China

**Keywords:** diamond powder, filling quantity, sodium silicate, thermal conductive adhesive, bonding properties, thermal conductivity

## Abstract

With the development of miniaturized, highly integrated, and multifunctional electronic devices, the heat flow per unit area has increased dramatically, making heat dissipation a bottleneck in the development of the electronics industry. The purpose of this study is to develop a new inorganic thermal conductive adhesive to overcome the contradiction between the thermal conductivity and mechanical properties of organic thermal conductive adhesives. In this study, an inorganic matrix material, sodium silicate, was used, and diamond powder was modified to become a thermal conductive filler. The influence of the content of diamond powder on the thermal conductive adhesive properties was studied through systematic characterization and testing. In the experiment, diamond powder modified by 3-aminopropyltriethoxysilane coupling agent was selected as the thermal conductive filler and filled into a sodium silicate matrix with a mass fraction of 34% to prepare a series of inorganic thermal conductive adhesives. The thermal conductivity of the diamond powder and its content on the thermal conductivity of the adhesive were studied by testing the thermal conductivity and taking SEM photos. In addition, X-ray diffraction, infrared spectroscopy, and EDS testing were used to analyze the composition of the modified diamond powder surface. Through the study of diamond content, it was found that as the diamond content gradually increases, the adhesive performance of the thermal conductive adhesive first increases and then decreases. The best adhesive performance was achieved when the diamond mass fraction was 60%, with a tensile shear strength of 1.83 MPa. As the diamond content increased, the thermal conductivity of the thermal conductive adhesive first increased and then decreased. The best thermal conductivity was achieved when the diamond mass fraction was 50%, with a thermal conductivity coefficient of 10.32 W/(m·K). The best adhesive performance and thermal conductivity were achieved when the diamond mass fraction was between 50% and 60%. The inorganic thermal conductive adhesive system based on sodium silicate and diamond proposed in this study has outstanding comprehensive performance and is a promising new thermal conductive material that can replace organic thermal conductive adhesives. The results of this study provide new ideas and methods for the development of inorganic thermal conductive adhesives and are expected to promote the application and development of inorganic thermal conductive materials.

## 1. Introduction

With the continuous progress of science and technology, the development of microelectronics technology has advanced at a rapid pace. The problem of heat dissipation of electronic devices has become a problem that restricts the development of microelectronics [1]. Since there is a gap between the heat sink and the electronic components, and the thermal conductivity of air is poor, thermal interface materials are needed to fill the gap between the electronic devices and the heat sink to enhance their thermal conductivity.

Xiangli Liu [2] used sodium silicate as a matrix and BN as a filler to prepare thermal conductive adhesives with thermal conductivity up to 3.52 W/(m·K). Meng et al. [3] prepared a composite adhesive using epoxy resin and graphene sheets. When the filler content was 6%, the thermal conductivity was 2.13 W/(m·K), and when the filler content was 0.375%, the lap shear strength was 13.57 MPa. Aradhana Ruchi et al. [4] prepared an epoxy-based thermal adhesive using poly-p-phenylene and graphene oxide as composite fillers. When the mass ratio of epoxy resin to graphene oxide was 10:1, the optimal thermal conductivity of the adhesive was 0.9 W/(m·K). Kumar et al. [5] prepared an epoxy-based thermal adhesive using carbon-based materials as thermal conductivity enhancers. When 40% of silicon-treated expanded graphite flakes were added, the thermal conductivity of the epoxy-based adhesive increased 17 times to 3.21 W/(m·K). In Fuqiang Tian [6], the problem of low thermal conductivity and electrical insulation properties of epoxy resin-based thermal conductive adhesives with high filler fillers was investigated by modifying the thermal conductivity fillers and compounding the fillers. Yunlai Guan, Zhenzhong Zhang et al. [7] investigated the effects of particle size and compounding ratio of h-BN powder on the thermal conductivity of epoxy resin-based thermal conductive adhesives. Gojny et al. [8] studied the thermal conductivity of epoxy resin as a matrix and multi-walled carbon nanotubes and single-walled carbon nanotubes as fillers. Jittiwat et al. [9] studied the thermal conductivity of polycarbonate as a matrix and carbon fibers with two aspect ratios of 40 μm and 100 μm as fillers. It was found that fillers with larger aspect ratios were more effective in improving the thermal conductivity.

JungsooKim [10] examined the effect of filler incorporation on the thermal and mechanical properties of epoxy composites.

Traditional thermal conductive adhesive is mainly organic matrix, but due to the poor thermal conductivity of organic matrix, poor high temperature resistance and easy aging problems limit its application, while sodium silicate, having the advantages of good anti-aging performance and relatively good thermal conductivity [2], is a new generation of thermal conductive adhesive matrix of choice. For the study of fillers, more scholars focus on materials such as boron nitride and alumina, and less relevant research has been done on diamond as a thermally conductive filler. Diamond is a three-dimensional mesh structure, and its carbon atoms have a tetravalent state, i.e., sp3 hybridized state, where each carbon atom is connected to four neighboring carbon atoms, sharing four pairs of valence electrons and forming four covalent bonds with its surrounding atoms. Its thermal conductivity comes from the lattice vibrations; thanks to the smaller mass of the element carbon and the stronger C-C bonds, the carbon atoms vibrate basically around the very small values of potential energy, hence the high thermal conductivity of diamond. With a thermal conductivity of 2000 W/(m·K), diamond is the best material known for its thermal conductivity [11]. Diamond also has good thermal stability, low coefficient of thermal expansion and other outstanding performance [12]. Some studies have shown that the bonding properties of thermal conductive adhesives increase with increasing filler content. However, the bonding performance eventually decreases after the optimum filler level is reached [13]. The filler content also affects the thermal conductivity of the adhesive, so it is important to balance the adhesive and thermal conductivity of the adhesive by adjusting the filler content.

Currently, the thermal conductivity of organic adhesives is limited and difficult to significantly improve, which hinders the development of the electronics industry. In this study, we investigated a thermal conductive insulation adhesive system that combines high thermal conductivity and strong adhesion. We innovatively proposed a diamond-reinforced sodium silicate-based high thermal conductivity thermal conductive adhesive system, which overcomes the interface limitations of filled thermal conductive organic adhesives. We verified the feasibility of using inorganic materials in the matrix of thermal conductive insulation adhesives, providing a practical and significant new idea for the effective application of diamond in the thermal conductive field. This study also has reference value for the development and progress of thermal interface materials.

## 2. Materials and Methods

### 2.1. Experimental Materials and Equipment

Experimental raw materials: diamond powder (Zhongyuan Superabrasives Co., Ltd., Shangqiu, China, particle size 1 μm), sodium silicate solution (Shandong Yousuo Chemical Technology Co., Ltd., Linyi, China, modulus 3.3, mass fraction 34%), 3-aminopropyltriethoxysilane (Changzhou Runxiang Chemical Co., Ltd., Changzhou, China), glacial acetic acid (Xi’an Jinxiang Pharmaceutical Excipients Co., Ltd., Xi’an, China, AR), ammonia (Fuchen Chemical Reagent Co., Ltd., Tianjin, China, mass fraction 25%), and anhydrous ethanol (Tianjin Fuyu Fine Chemical Co., Ltd., Tianjin, China, AR).

Experimental equipment: Blast drying oven (101-1A, Beijing Zhongxingweiye Instruments Co., Ltd., Beijing, China), Vacuum Tube Furnace (M1210, Henan Cheng Yi Equipment Technology Co., Ltd., Zhengzhou, China), ultrasonic cleaner (SB-80, Ningbo Xin Yi Biological Technology Co., Ltd., Ningbo, China), water bath (DZKW-C, Henan Wolin Instruments Co., Ltd., Zhengzhou, China), thermal conductivity tester (DM3615, Shanghai Dongmao Electronic Technology Co., Shanghai, China), microcomputer-controlled universal testing machine (WY-10TB, Suzhou Wan Yi Experimental Instruments Co., Ltd., Suzhou, China), Fourier transform infrared spectroscopy analyzer (FTIS-730, Shenzhen Junhuiteng Technology Co., Ltd., Shenzhen, China), scanning electron microscope (PHENOMPROX, Phenom Scientific Instrument (Shanghai) Co., Ltd., Shanghai, China), X-ray diffractometer (MINIFLEX600, Japan), benchtop centrifuge (AXTG16G, Yancheng Anxin Experimental Instruments Co., Ltd., Yancheng, China), and magnetic stirrer (HJ-3, Beijing Nengke Engineering Co., Ltd., Beijing, China).

### 2.2. Diamond Powder Coupling Treatment

As diamond has hydroxyl functional groups on its surface, the surface can be modified using silane coupling agents. This allows it to bond more firmly with the matrix and also enhances the dispersion of the diamond powder in the matrix [14], which facilitates better formation of thermally conductive pathways for the filler in the sodium silicate matrix. In this experiment, 3-aminopropyltriethoxysilane was used to modify the surface of diamond powder.

According to the chemical bonding theory, the reaction mechanism between the organosilane coupling agent molecules and the nanodiamond surface can be formulated as follows:

(1) Hydrolysis of organosilanes to silanol:

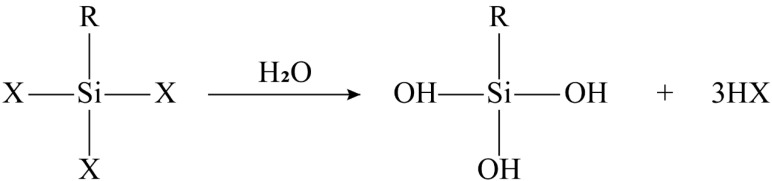

(2) The silanol is bonded to the diamond surface: first, the silanol is hydrogen bonded to the diamond surface:

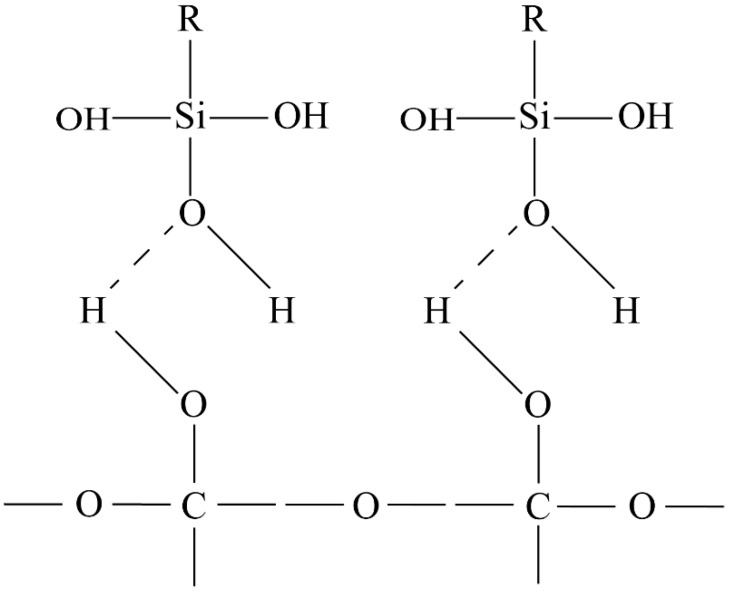

Then, hydrogen bonds are also formed between the coupling agent molecules, which are dehydrated to form Si-O-Si bonds, i.e., oligomers of silane coupling agents:

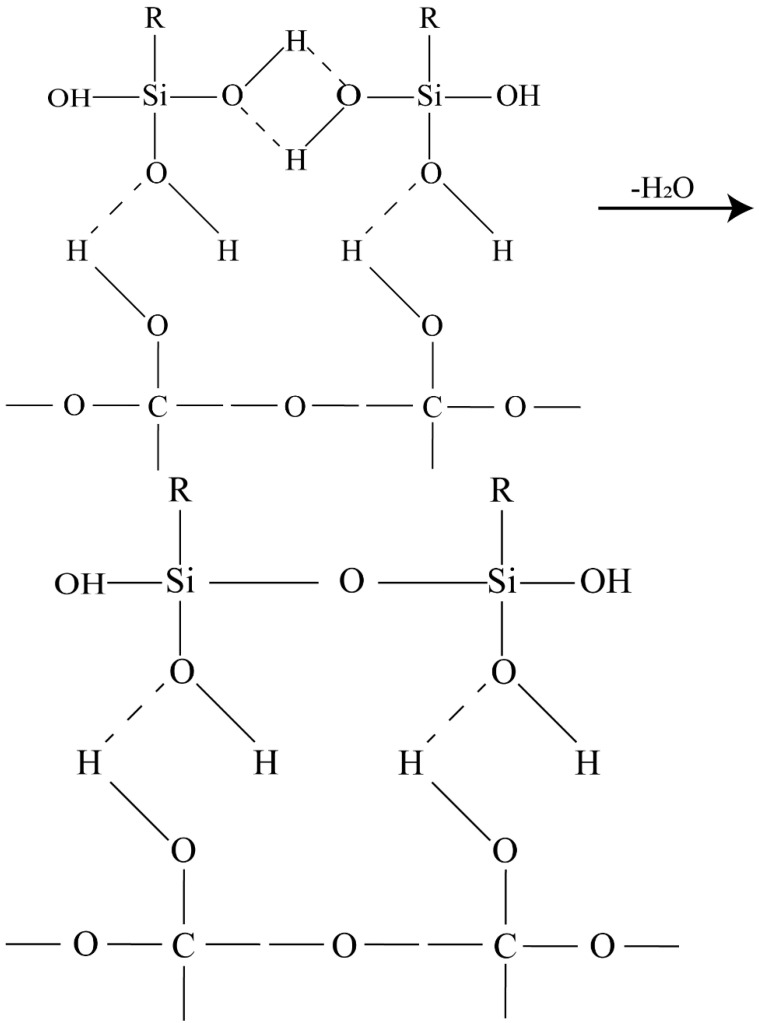



Finally, a dehydration reaction occurs to form partially covalent oxygen bridge bonds, which allows for the coverage on the surface of the filler:

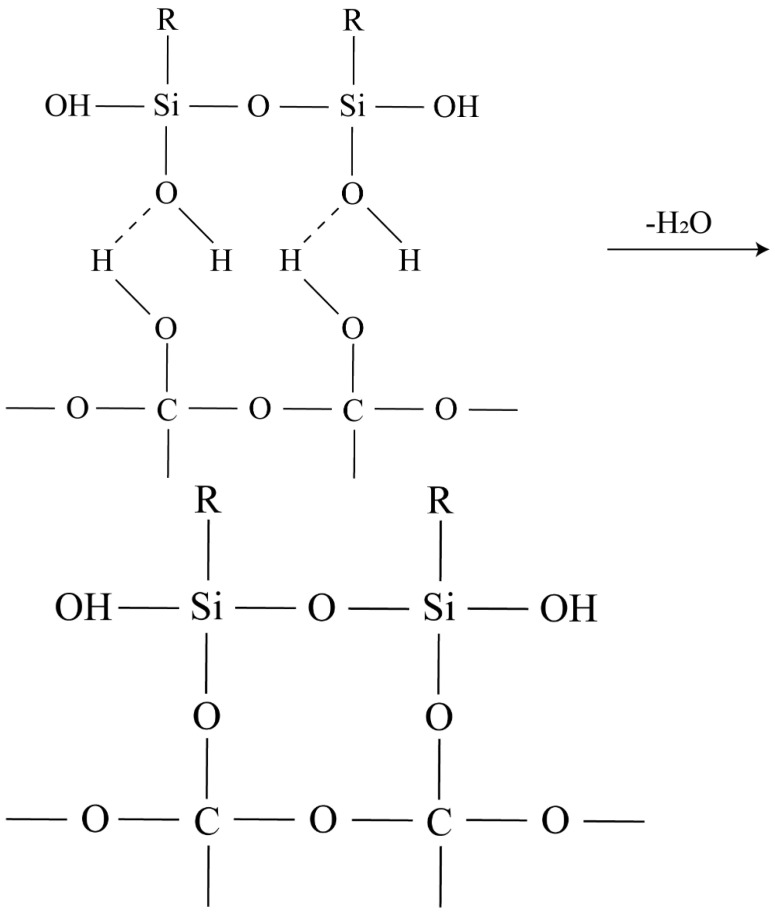



After mixing with the matrix, silane coupling agent molecules grafted on the surface of diamond micro-powders cross-link with the matrix molecules through hydrogen bonding and are attracted and approached to the matrix molecules through intermolecular forces such as electrostatic forces and van der Waals forces. Finally, they wrap around each other, forming a special interfacial layer between the diamond micro-powders and the matrix. The more functional groups on the surface of diamond micro-powders, the denser the interfacial layer formed and the greater the reinforcing effect.

The specific operation steps for surface modification of diamond micro-powders are as follows:

(1) Weigh 1 g of diamond powder with a balance and measure 30 mL of anhydrous ethanol with a measuring cylinder, mix, add to a three-necked flask, and stir the sample at room temperature to disperse the diamond powder evenly in the anhydrous ethanol.
(2) Diamond micro powder is measured according to the solid–liquid ratio of 5:1 mL/g with the silane coupling agent, and the silane coupling agent is mixed with water according to the ratio of 1:2.
(3) Pour the mixed solution of silane coupling agent and water into the mixed solution of diamond micro powder and anhydrous ethanol, and stir thoroughly to mix it well.
(4) Condensation reflux reaction in a constant temperature magnetic stirrer at 70 °C for 3 h.
(5) After the reaction is completed, the reaction is repeatedly washed with anhydrous ethanol and filtered by extraction until the filtrate is clear and transparent, and the filter cake is placed in a vacuum drying oven and dried at 70 °C to obtain modified diamond particles.

### 2.3. Preparation of Sodium Silicate-Based Diamond Thermal Conductive Adhesive Specimens

The mass fraction of sodium silicate chosen here was 34% and the remaining 66% was mainly water. Because the sodium silicate solution is extremely miscible with water and the water in the gum solution also evaporates very easily during mechanical stirring, the choice here was made to match the sodium silicate solute to the diamond micro powder by mass. If the solute mass is 34 g in 100 g of sodium silicate, when 34 g of diamond is added, the diamond micro powder will account for 50% of the overall dry weight mass. All mass fractions of diamond fines below are expressed as a percentage of dry weight mass.

(1) The disc-shaped graphite mold is coated with liquid paraffin and placed in a blast drying oven for 3 h at 80 °C.
(2) Prepare colloids with different filling contents using diamond powder as filler as shown in Table 1. Stirred magnetically for 1 h, poured into the treated disc mold, then placed in a drying oven and cured in the oven at a temperature of 50 °C for 1 h, cooled and demolded.

### 2.4. Performance Testing and Characterization

The surface morphology of the diamond powder before and after treatment with silane coupling agent and the cross-sectional morphology of the thermal conductive adhesive with different diamond fillings were characterized by scanning electron microscopy; the physical phase composition of the diamond powder, sodium silicate, and thermal conductive adhesive used were tested by X-ray diffractometer; the bonding performance of the thermal conductive adhesive was tested by a material universal testing machine; the thermal conductivity of the thermal conductive adhesive was tested by a thermal conductivity tester.

## 3. Results and Discussion

### 3.1. SEM Characterization and Analysis

(1) Surface morphology of diamond powder before and after surface modification

**Figure 1:**
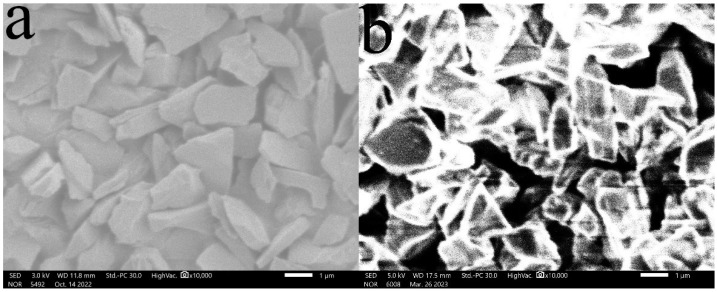
Surface morphology of diamond powder before and after silane coupling agent modification: (**a**) before modification; (**b**) after modification.

**Figure 2:**
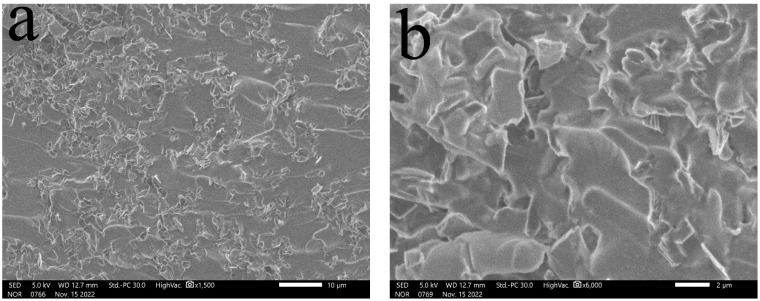

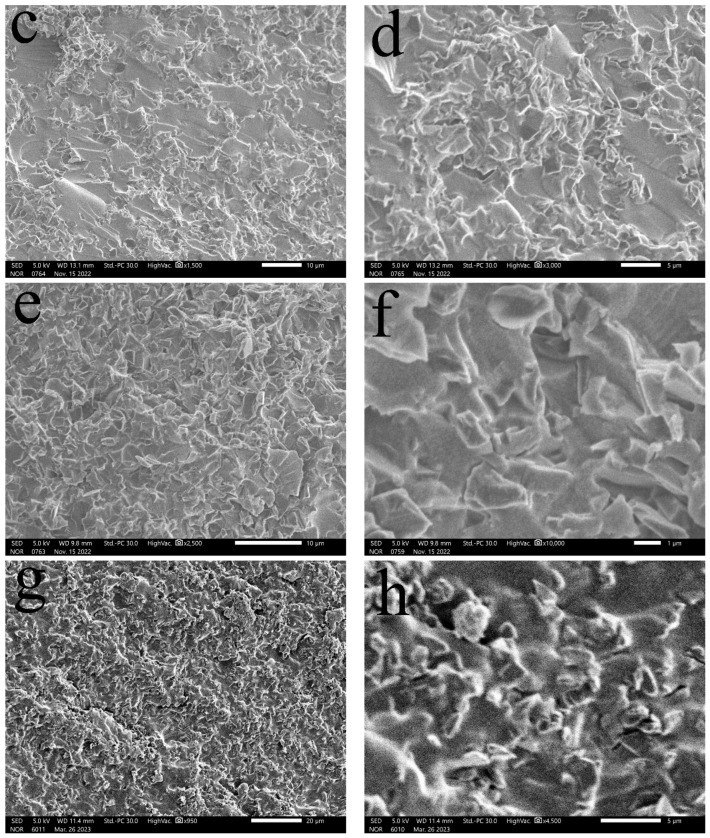
Cross-sectional profiles of sodium silicate-based thermal conductive adhesives with different diamond micronaire fillings: (**a**,**b**) are mass fractions of 10%; (**c**,**d**) are mass fractions of 30%; (**e**,**f**) are mass fractions of 50%; (**g**,**h**) are mass fractions of 80%.

Figure 1a shows the surface morphology of the diamond powder before the surface modification with 3-aminopropyltriethoxysilane, which shows that the surface is clean and free of impurities, and Figure 2b shows the surface morphology of the diamond powder after the surface modification with 3-aminopropyltriethoxysilane, which shows that a layer of silane coupling age t is clearly and uniformly attached to the surface.

(2) Distribution of diamond powder with different content in the matrix

Figure 2a–h show the cross-sectional morphology of the cured sodium silicate based thermal conductive adhesive at 10%, 30%, 50%, and 80% mass fraction of diamond micronaire, respectively. It can be seen that at 10% diamond powder mass fraction, the diamond powders are independent of each other and have an island-like structure, with no contact between the fillers and no good thermal pathway. When the mass fraction of diamond powder is 30%, the fillers are basically in contact with each other and a certain thermal pathway has been formed, but some of the fillers are not in direct contact with each other and there is still a layer of sodium silicate matrix, which indicates that its content can be further increased. When the mass fraction of diamond powder is 50%, the probability of mutual contact between the fillers is greater, and more effective heat conduction pathways have been formed, and it can be seen that the bond between diamond and matrix is better. When the mass fraction of diamond powder reaches 80%, there are too few matrix materials to fill the gaps between the fillers, and many voids appear between the fillers. Since air has poor thermal conductivity, this structure will severely affect the thermal conductivity of the thermal adhesive. In addition, due to insufficient matrix connections between the fillers, the adhesion performance of the thermal adhesive will also be significantly weakened.

### 3.2. EDS Characterization and Analysis

**Figure 3:**
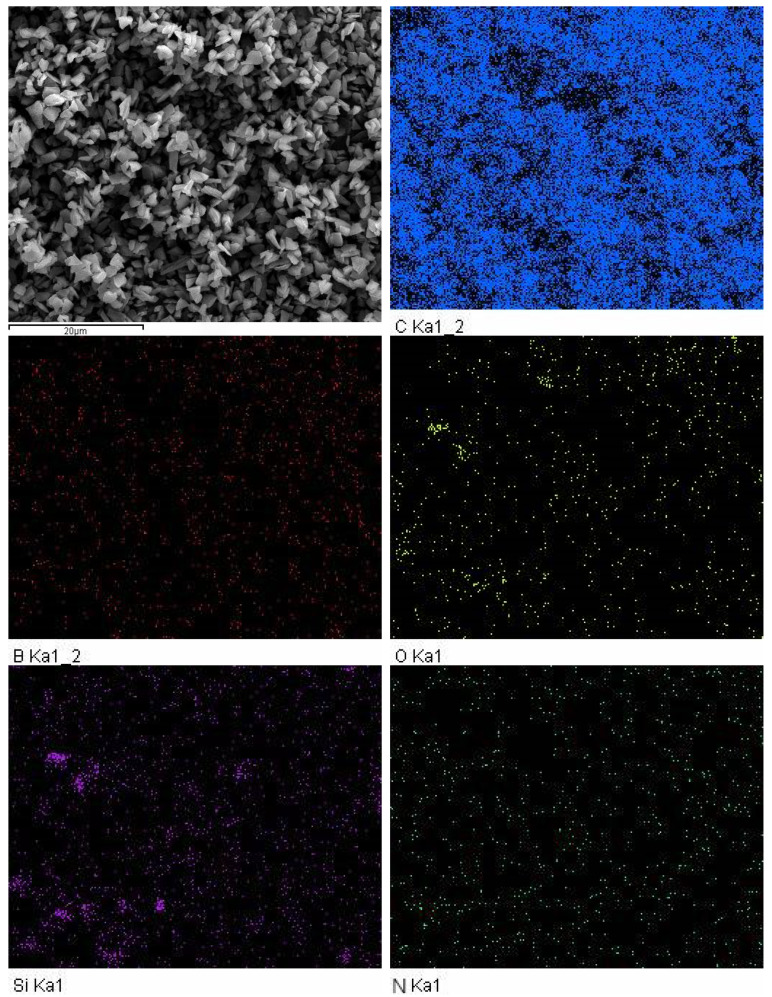
EDS image of diamond powder after modification with silane coupling agent.

After modification of diamond micro-powder with the silane coupling agent 3-aminopropyltriethoxysilane, the EDS spectrum showed changes. As shown in Figure 3, the surface distribution of elements on the diamond micro-powder after modification with the silane coupling agent was uniform, including five elements of C, B, O, Si, and N, with weight percentages of 91.67%, 5.29%, 1.83%, 0.56%, and 0.65%, respectively. After hydrolysis, the silane coupling agent 3-aminopropyltriethoxysilane combined with the hydroxyl groups on the surface of the diamond micro-powder, forming a layer of silane coupling agent film, and the dehydrated condensation reaction led to the coating of the silane coupling agent 3-aminopropyltriethoxysilane on the surface of the diamond micro-powder. Among them, the B element comes from diamond, the C element comes from diamond and silane coupling agent, and the O, Si, and N elements come from the silane coupling agent. The EDS spectrum only showed the presence of the silane coupling agent and diamond elements, without any other impurities, indicating that there was only silane coupling agent on the surface of the diamond micro-powder, and no other impurities were present.

### 3.3. XRD Characterization and Analysis

**Figure 4:**
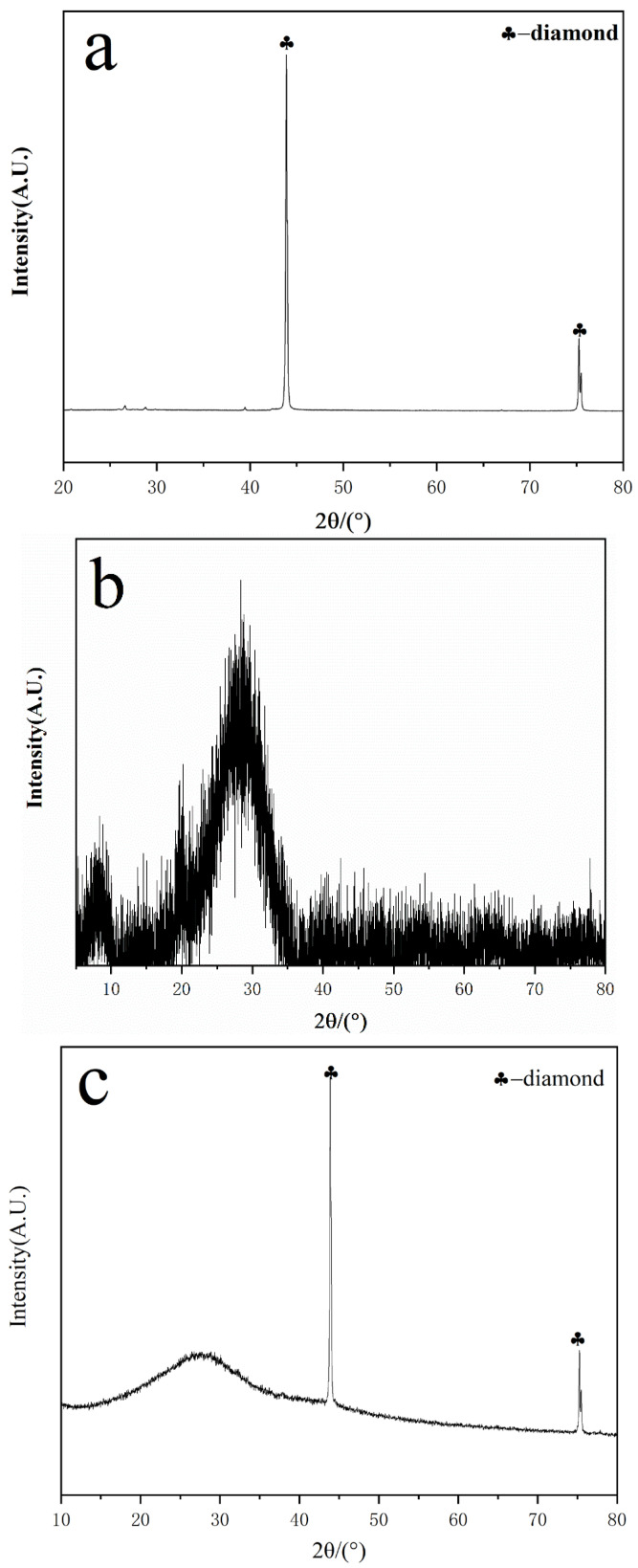

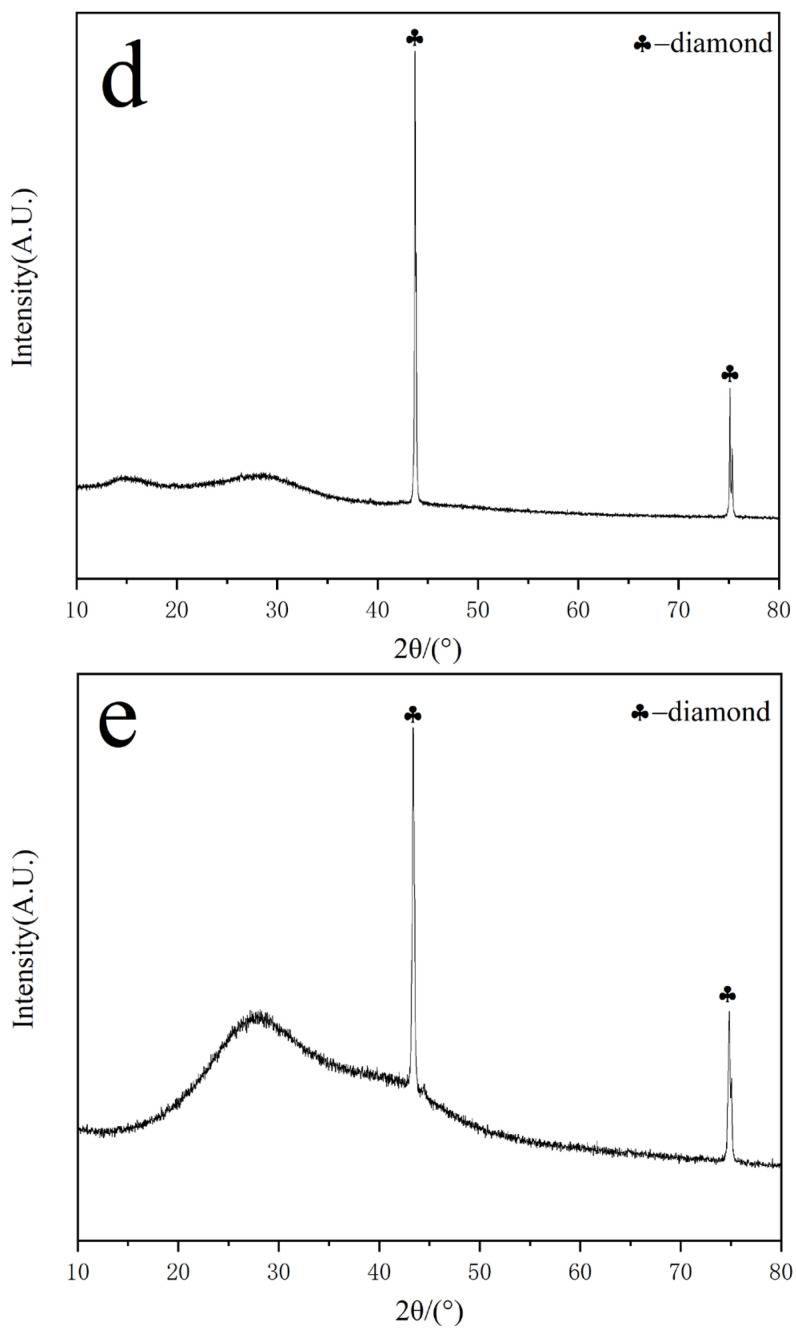
XRD patterns of diamond powder, sodium silicate, and sodium silicate-based diamond thermal conductive adhesive: (**a**) Diamond powder; (**b**) sodium silicate; (**c**) thermal conductivity adhesive with 30% diamond mass fraction; (**d**) thermal conductivity adhesive with 50% diamond mass fraction; (**e**) thermal conductivity adhesive with 80% diamond mass fraction.

Figure 4a is the XRD pattern of diamond powder as a filler, Figure 4b is the XRD pattern of sodium silicate used in this experiment; there is a typical “bun peak” at the low angle end and no other impurity phases are present [15]. Figure 4c is the XRD pattern of the thermal conductivity adhesive with 30% diamond mass fraction, Figure 4d is the XRD pattern of a thermal conductive adhesive with 50% diamond mass fraction, and Figure 4e is the XRD pattern of a thermal conductive adhesive with 80% diamond mass fraction. As can be seen in Figure 4c–e, the XRD patterns of diamond thermal conductive adhesives with different mass fractions are basically the same. In the thermal adhesive, the characteristic diffraction peak position of diamond remains unchanged, and the peak shape is regular, indicating that diamond is only added as a filler to the sodium silicate matrix and no other reaction occurs. The “bun peak” of sodium silicate at the low angle remains unchanged, indicating that no new phases were formed or impurities were introduced during the preparation of the thermal conductive gel.

### 3.4. Characterization and Analysis of Bonding Properties

**Figure 5:**
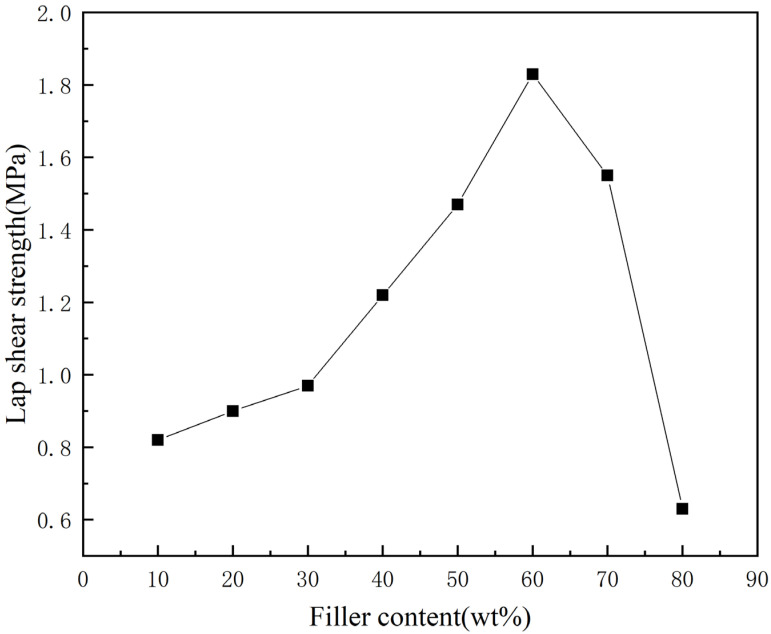
Effect of different diamond micron content on the tensile shear strength of sodium silicate-based thermal conductive adhesives.

The tensile shear strength of the thermal conductive adhesive was determined according to the GB/T7124-2008 method for the determination of tensile shear strength of adhesives [16]. The thermal conductive adhesive was applied to a standard sample, allowed to air dry, and then the standard sample was closed and pressed to cure, resulting in a well-bonded sample. The tensile shear strength curve of the adhesive was obtained by measuring five times at a stretching speed of 20 mm/min on an electronic universal testing machine WY-10TB and taking the average value. It can be seen from Figure 5 that the tensile shear strength of the thermal conductive adhesive increases slowly with the increase of diamond content and reaches the maximum when the diamond mass fraction increases to about 60%; when the diamond content continues to increase, the tensile shear strength of the thermal conductive adhesive starts to decrease, especially when the diamond mass fraction is 70–80%, the tensile shear strength of the thermal conductive adhesive decreases rapidly. This is because when the diamond content is low, the sodium silicate matrix is combined with the diamond, and the diamond as the reinforcing particle is combined with the matrix, which can effectively improve the strength of the thermal conductive adhesive; the more the diamond filling, the better the reinforcing effect, and the bonding performance of the thermal conductive adhesive is gradually improved. When the bonding performance reaches the optimum, then add diamond. Because the content of the matrix becomes relatively small, diamond and matrix cannot be effectively combined together, while too much diamond destroyed the continuity of the sodium silicate matrix, forming a large number of gaps, greatly reducing the diamond and sodium silicate contact area. At this time, the thermal conductive adhesive has a poor bonding performance. Therefore, the diamond content has an optimum value to improve the adhesive performance of the thermal conductive adhesive, too little diamond content will not have a great improvement to the adhesive performance of the thermal conductive adhesive, too much diamond content will make the adhesive performance of the thermal conductive adhesive decline. The tensile shear strength at 60% diamond content is 1.83 MPa, which is nearly double that of other products in the same industry and can meet the requirements of use.

### 3.5. Characterization and Analysis of Thermal Conductivity

The prepared thermal conductive adhesive was placed in a cylindrical mold with a height of 7 mm and a diameter of Φ 20 mm. After being left at room temperature for two days, it was placed in a drying oven for low-temperature baking. The cured adhesive was then removed from the mold, and its upper and lower surfaces were polished smoothly to make the two surfaces parallel. To meet the instrument requirements, the thickness of the polished sample was controlled within 5 mm. The DM3615 thermal conductivity tester was used to test the sample, and then the measured values were compared and analyzed.

**Figure 6:**
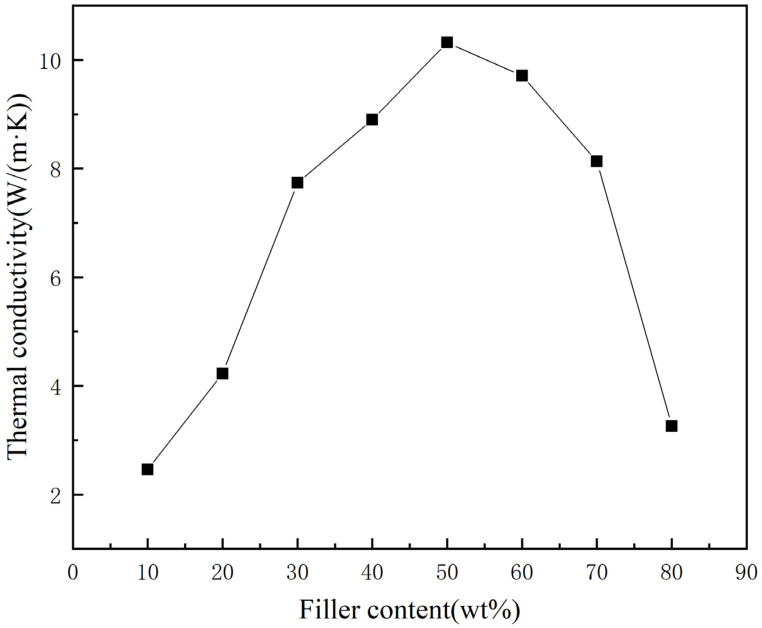
Effect of different diamond micron content on the thermal conductivity of sodium silicate-based thermal conductive adhesives.

From Figure 6, it can be seen that the thermal conductivity of the thermal conductive adhesive increased first with the increase of diamond content, reaching the highest at a diamond mass fraction of 50% at 10.32 W/(m·K); when the diamond content continued to increase, the thermal conductivity of the thermal conductive adhesive started to decrease. This is because when the diamond filler is too small, most of the thermal conductive adhesive is occupied by the sodium silicate matrix, and it is difficult to form an effective thermal conductivity path between the diamonds. As the diamond content increased, more and more contact occurred between the diamonds and they were well bonded together by the sodium silicate matrix, and the thermal conductivity started to improve; especially when the diamond mass fraction was 20–30%, the thermal conductivity increased rapidly. After the thermal conductivity reaches its maximum value, as the diamond content continued to increase, although a large number of thermally conductive networks were formed inside the thermal conductive adhesive, it was difficult to fill all the gaps between the diamonds because the sodium silicate matrix content gradually decreased, and a large number of air gaps were created inside the thermal conductive adhesive. The thermal resistance of the interface between the fillers rose, and the thermal conductivity of the thermal conductive adhesive began to decline.

## 4. Conclusions

As electronic technology continues to advance, electronic products are becoming more powerful and high-performance, but this also means that the heat generated per unit area is increasing rapidly. This poses higher requirements for heat management of electronic products because high temperatures can affect the performance and lifespan of electronic products, and even lead to damage. Currently, the heat management system still has a long way to go before achieving high integration. Therefore, the heat conduction and connection between various components heavily relies on thermally conductive adhesives with high thermal conductivity and strong adhesion. In this paper, a novel composite thermally conductive adhesive is proposed with sodium silicate as the matrix and diamond powder as the thermal conductive filler. A thermally conductive adhesive with high thermal conductivity is prepared and characterized for its basic properties and performance. The main conclusions obtained in this paper are as follows:

(1) At low filler levels, the bonding performance of thermal conductive adhesives can be effectively improved with the increase of diamond content. After that, with the increase of diamond content, the bonding performance of thermal conductive adhesive starts to decrease, especially when the mass fraction of diamond is 70–80%, the bonding performance of thermal conductive adhesive decreases sharply.
(2) At low filling levels, the thermal conductivity of the thermal conductive adhesive gradually increases as the diamond content increases, and when the diamond mass fraction reaches 20–30%, the thermal conductivity increases rapidly, and the thermal conductivity reaches its highest value of 10.32 W/(m·K) at 50% diamond mass fraction. After that, the thermal conductivity of the thermal conductive adhesive starts to decrease as the diamond content increases.
(3) The suitable diamond mass fraction for this thermal conductive adhesive is 50–60%. Within this range, there is an optimum value for the thermal conductivity and adhesive performance of the adhesive, and the diamond content can be adjusted within this range according to the different requirements for thermal conductivity and adhesive performance.

This research provides a new idea for the effective application of diamond in the field of thermal conductivity and has scientific guiding significance for the development of thermal interface composite materials. The thermal conductive adhesive prepared in this study can be applied to the assembly and installation of heat sinks for electronic products such as computers, mobile phones, and tablets, as well as the heat dissipation of engines and electronic control modules in the automotive industry, the heat dissipation of LED lights, etc. In short, it has a wide range of applications in fields where heat dissipation is required and can improve the reliability and stability of equipment.

## Figures and Tables

**Table 1:** Proportioning of the thermal conductive adhesives.

Diamond Grain Size (μm)	Diamond Powder as a Percentage of Overall Dry Weight (%)				
1	10	20	30	40	50

## Data Availability

Not applicable.
